# Sensitive SERS nanotags for use with a hand-held 1064 nm Raman spectrometer

**DOI:** 10.1098/rsos.170422

**Published:** 2017-07-19

**Authors:** Hayleigh Kearns, Fatima Ali, Matthew A. Bedics, Neil C. Shand, Karen Faulds, Michael R. Detty, Duncan Graham

**Affiliations:** 1 Department of Pure and Applied Chemistry, Technology and Innovation Centre, University of Strathclyde, 99 George Street, Glasgow G1 1RD, UK; 2 Department of Chemistry, University at Buffalo, The State University of New York, Buffalo, NY 14260, USA; 3 Dstl, Porton Down, Salisbury, Wiltshire SP4 0JQ, UK

**Keywords:** hand-held Raman spectrometer, short-wave infrared excitation, surface-enhanced Raman scattering nanotags, limits of detection, hollow gold nanoshells, chalcogenopyrylium dyes

## Abstract

This is the first report of the use of a hand-held 1064 nm Raman spectrometer combined with red-shifted surface-enhanced Raman scattering (SERS) nanotags to provide an unprecedented performance in the short-wave infrared (SWIR) region. A library consisting of 17 chalcogenopyrylium nanotags produce extraordinary SERS responses with femtomolar detection limits being obtained using the portable instrument. This is well beyond previous SERS detection limits at this far red-shifted wavelength and opens up new options for SERS sensors in the SWIR region of the electromagnetic spectrum (between 950 and 1700 nm).

## 1. Introduction

Raman spectroscopy is an efficient and effective technique for identifying and distinguishing between materials [1]. It offers good molecular specificity and, due to recent advancements in instrumentation, is regarded as a portable and easy-to-use technique [2]. In addition, there is little to no sample preparation required, it is non-contact and non-destructive and as such is currently being employed in a range of industries from pharmaceutical to counter-terrorism and from art to archaeology [1–5]. Traditionally, Raman spectroscopy employed laser excitation wavelengths in the visible region because the intensity of Raman scattering is dependent on the fourth power of the excitation frequency. Thus, the scattering effect is stronger at shorter wavelengths (532–785 nm). However, at visible excitation wavelengths, problems can be encountered such as increased background fluorescence and sample degradation [1]. The number of molecules which fluoresce at shorter wavelengths is greater than at longer wavelengths. Hence, by moving to a short-wave infrared (SWIR) laser excitation between 950 and 1700 nm, fluorescence can be significantly reduced [6–8]. In addition, there is limited photobleaching and the infrared region provides an uncongested spectral window for optical analysis due to the absorption and scattering backgrounds of many molecules (in particular, bio-molecules) being at a minimum [9–12]. In order to use these advantages, research focused on the design of Raman instruments operating at these longer wavelengths and instruments that are portable are of particular current interest [6,13]. As a result, there has been an increase in the number of publications reporting the use of portable 1064 nm Raman spectrometers with the main areas of interest involving homeland security, forensic, biomedical and geological applications [2–4,14,15].

Raman scattering is significantly weaker in the infrared region than in the visible and as such, weak signals are often obtained. Nonetheless, the poor signal response can be overcome by attaching or trapping molecules close to the surface of metallic nanoparticles, thus giving rise to the phenomenon known as surface-enhanced Raman scattering (SERS) [1,16]. The intensity of scattering from SERS is dependent on coupling with the plasmon from the enhancing surface and can give intense signals well into the infrared [17,18]. Enhancements of 10^6^ have been reported, thus are several orders of magnitude greater than those reported for conventional Raman scattering [19–21]. Additional enhancements, up to 10^14^, can be achieved by tuning the laser frequency with an electronic transition within the analyte hence using a chromophore bound to a roughened metal surface to give rise to the phenomenon known as surface-enhanced resonance Raman scattering (SERRS) [1]. Therefore, the SERRS enhancement is due to both surface plasmon resonance and molecular resonance and it allows for greater enhancement factors to be obtained while also improving the sensitivity and selectivity [22].

SERS nanotags are *in situ* probes consisting of metallic nanoparticles and organic reporter molecules. They provide sensitive and selective analytical tools for studying chemical and biological systems [23]. Aggregated noble metal nanoparticles, commonly silver and gold, are used as suspension-based SERS substrates, as they are stable materials and have localized surface plasmon resonances (LSPR) that can be tuned from the visible to the infrared region by modifying the size, shape or surface chemistry [23,24]. To date, most of the research has focused on designing SERS nanotags for use with visible laser excitations but due to the benefits of operating in the SWIR region, research is emerging on the use of SERS combined with 1064 nm laser excitation and very recently with 1280 and 1550 nm [25–30].

To coincide with advancements in instrumentation, it is vital to develop SERS nanotags that are capable of providing sensitive and reproducible responses in the uncongested spectral window of the infrared region while using a portable device. Up till now, the majority of publications have reported the use of FT-Raman spectrometers; however, recently reports are emerging with the use of dispersive Raman instruments [25–27]. Although these instruments are technically classed as portable, they are bulky, heavy units which still require connection to a power supply and computer. Hand-held instruments, on the other hand, are unique in that they are small, light, battery-operated (if required) and can be easily used by a single operator in diverse and challenging environments. We believe that a hand-held Raman spectrometer in combination with our unique chalcogenopyrylium nanotags could provide the portability and sensitivity required to combat the challenges currently being faced in the SWIR region, and thus could be pivotal for future advancements in homeland security, biological and clinical applications.

Herein, we report that hollow gold nanoshells (HGNs) modified with chalcogenopyrylium dyes give strong SERS responses with femtomolar detection limits being obtained using a hand-held 1064 nm Raman spectrometer. The Snowy Range CBEx Raman spectrometer is regarded as hand-held, light-weight and portable as it has the following dimensions 4.5 × 3.125 × 2.25 inches, weighs 773 g and can operate solely using four AA batteries. We have previously demonstrated the SERS capabilities of the chalcogenopyrylium dyes with HGNs and large gold nanoparticles; with picomolar (pM) detection limits being obtained using both 1280 and 1550 nm laser excitations [28,29]. It was found that 2-thienyl and 2-selenophenyl substituents make excellent attachment groups for adsorbing strongly onto gold surfaces and the fine tuning of the absorbance maxima into the infrared region arises by interchanging the chalcogen atoms in the backbone and in the ring systems.

Specifically, in this work, the SERS capabilities of HGNs functionalized with 17 different chalcogenopyrylium dyes (chalcogen nanotags) were investigated using a hand-held 1064 nm Raman spectrometer. It should be noted that an inorganic salt, specifically potassium chloride (KCl), was used to aggregate the nanotags as it increases the SERS signal by screening the Coulombic repulsion energy between the nanoparticles allowing the reporter molecules to adhere more closely to the nanoparticle surface. We have previously shown that reproducible and stable nanotags (HGNs functionalized with a Raman reporter) can be prepared when 30 mM KCl is used as the aggregating agent [28,31]. It was found at a concentration greater than 30 mM, the HGNs precipitated out of solution and below this, weaker SERS signals were observed. Moreover, we demonstrated that by using this optimal concentration of KCl, the LSPR of the HGNs did not shift, but the enhancement in SERS signal was undeniable. Extinction spectroscopy, dynamic light scattering and zeta potential analysis were used previously to investigate the stability of the nanotags and it was found that for both the commercial reporter BPE (1,2-bis(4-pyridyl)ethylene) and chalcogenopyrylium dye 14, that upon adding the Raman reporter and aggregating with KCl, the LSPR of the HGNs did not shift nor was there broadening of the peak; however, slight dampening in the absorption maxima was observed. Further, a size increase and a decrease in zeta potential values indicated a change to the colloidal solution, but ultimately, it was concluded that the nanotags were stable and not over-aggregating [28,31]. In addition, it is important to note that the plasmon resonance frequencies, hence LSPR of the HGNs (which is 710 nm, electronic supplementary material, figure S1), do not need to match the excitation frequency of the laser for effective SERS to be achieved [31–34]. Therefore, by exploiting our previous knowledge, SERS nanotags which are optimal for use in the SWIR region have been developed. Moreover, in this work, the SERS response from chalcogen nanotags were compared with commercial reporters BPE and AZPY (4,4-azopyridine) also adsorbed onto the surface of HGNs and aggregated with 30 mM KCl. BPE and AZPY were chosen as they are non-resonant commercial reporters which have previously been exploited and shown to provide excellent SERS responses with HGNs and other SERS substrates at this laser wavelength [31,35–37].

Dyes 1–17 incorporate sulfur and selenium atoms in the chalcogenopyrylium core and, as previously mentioned, the 2-thienyl and 2-selenophenyl substituents on select members of this library provide unique attachment points for adsorbing onto the gold surface of the HGNs [28]. It has previously been reported that dye 14 binds to the HGN surface with a chalcogen tripod arrangement, essentially with two selenium atoms and one sulfur atom directly facing the gold surface [17]. SERS, SERRS, theoretical calculations and sum-frequency generation vibrational spectroscopy were all used to define the orientation and manner of attachment. The study was very extensive in which the interpretation of the SE(R)RS spectra was thorough, with each peak being assigned and the changes in the spectra (between the laser wavelengths) being explained in terms of the SERS effect. A study of this scale is not possible for all the nanotags tested herein; however, general observations have been made and the results obtained support our previous findings.

**Figure 1.**
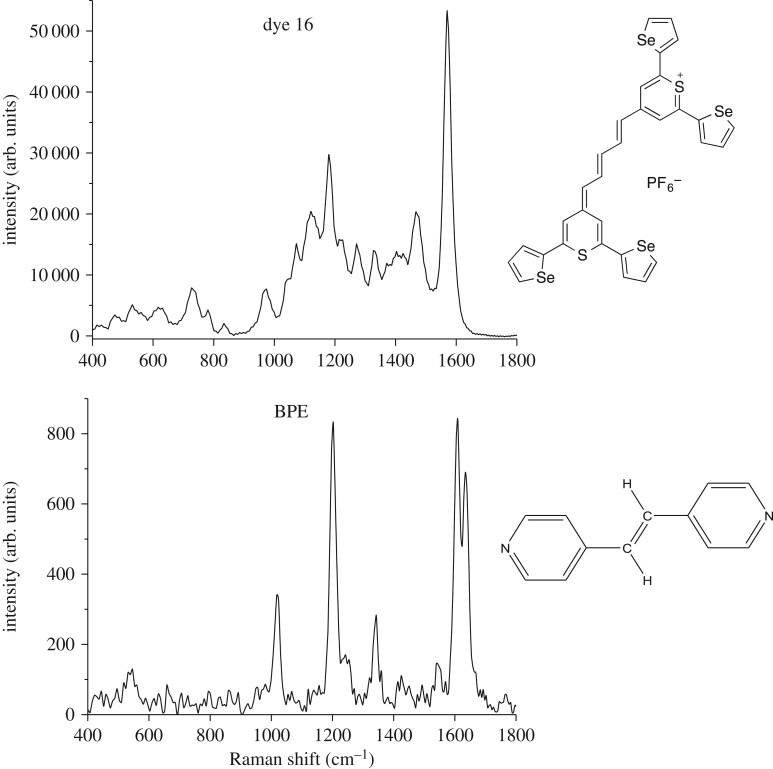
Comparison of SERS spectra for chalcogenopyrylium dye 16 with the commercial Raman reporter BPE. The dyes with a concentration of 10 µM were analysed with HGNs (SPR recorded at 710 nm; electronic supplementary material, figure S1) and KCl. A laser excitation of 1064 nm was employed in this analysis with an exposure time of 0.05 s for the chalcogen dye 16 and 1 s for the commercial reporter BPE. All spectra have been background corrected.

It can be observed in figure 1 and electronic supplementary material, figure S3 that modification of the dye substituents alters the SERS spectrum significantly. The structures for each of the chalcogen dyes plus the commercial reporters are given in electronic supplementary material, figure S2. Dyes 1–17 are highly aromatic with absorbance maxima from 653 to 986 nm (table 1). The HGNs have an LSPR at 710 nm (electronic supplementary material, figure S1) and it was found that the dyes attach strongly onto the surface of HGNs and as such produce vibrationally rich and intense SERS spectra using the 1064 nm laser excitation (figure 1; electronic supplementary material, figure S3). In general, the dyes which have absorption maxima closest to the laser excitation wavelength produced the best SERS signals, but this was to be expected as contributions from both molecular resonance and surface enhancement would have led to the increased SERS response [1]. Experimental details on the SERS characterization of the nanotags and synthetic scheme of the new chalcogenopyrylium dye 15 are provided in the Material and methods section with additional information being provided in the electronic supplementary material.

**Table 1.** Values of the absorption maximum (*λ*_max_) and calculated LOD values from the SERS experiment with associated s.d. error for 17 chalcogenopyrylium dyes plus BPE and AZPY adsorbed onto HGNs using a hand-held 1064 nm Raman spectrometer.

dye	*λ*_max_ nm (CH_2_Cl_2_)	1064 nm LOD, pM^a^	structure type
1	653	0.7 ± 0.07	monomethine dyes
2	676	0.8 ± 0.08	
3	699	0.7 ± 0.10	
4	676	1.0 ± 0.12	
5	698	0.6 ± 0.05	
6	723	1.1 ± 0.16	
7	659	0.6 ± 0.05	
8	687	0.5 ± 0.08	
9	806	0.6 ± 0.05	trimethine dyes
10	784	0.3 ± 0.03	
11	810	0.3 ± 0.02	
12	789	0.3 ± 0.02	
13	813	0.2 ± 0.02	
14	826	0.1 ± 0.01	
15	748	1.0 ± 0.12	
16	959	4.6 ± 0.34 fM*	pentamethine dyes
17	986	4.8 ± 0.37 fM*	
18—AZPY	non-resonant	0.15 ± 0.02 nM*	commercial reporters
19—BPE	non-resonant	0.19 ± 0.02 nM*	

^a^SERS—LOD ± s.d.

*highlight femtomolar and nanomolar concentrations.

Figure 1 and electronic supplementary material, figure S3 show the SERS spectra obtained when each of the reporter molecules at a concentration of 10 µM were added to the HGNs and analysed using a hand-held 1064 nm Raman spectrometer. All the measurements had a 0.05–1 s acquisition time and a laser power operating at 30 mW. It should be noted that the experimental set-up shown in electronic supplementary material, schematic S1*a* was employed in these studies. The experimental and instrumental parameters were kept constant throughout the analysis. In addition, Raman reporter and KCl stocks were freshly prepared daily. The solutions of each component (HGN, reporter, KCl) were tested separately as were the glass vials to ensure that no Raman signal was observed prior to analysis of the nanotag. Therefore, eliminating the chance of contamination and confirming that the signal observed was solely due to the SERS active nanotags. It should be noted that the nanotags were compared based on their intensities. Therefore, when comparing the most intense peak at approximately 1600 cm^−1^ which arises due to heterocyclic aromatic ring stretching within the molecule [17,28,36,37]; the pentamethine dyes (16 and 17) produced the strongest SERS response with the hand-held spectrometer, followed by the trimethine substituents, then the monomethine substituents and finally the commercial reporters produced the weakest SERS response. Generally, these results abide by the selection rules of SERRS; with the strongest signals being observed from the chalcogen dyes (dyes 16 and 17), which have absorption maxima closest to the laser frequency, and the poorest signals being observed from the non-resonant commercial reporters. The SERRS effect alone, however, could not be used to determine which nanotag would produce the best Raman signals as we have shown previously that the number of *sp*^2^ carbons present in the chalcogenopyrylium backbone and functional groups used for binding to the HGN surface all have an effect on the signal [28,29].

When comparing BPE with the strongest chalcogen dye, a signal enhancement of approximately 60-fold was observed for dye 16 over the commercial reporter (figure 1). The enhancement was even greater when comparing dye 16 to AZPY, thus demonstrating the superiority of these chalcogen dyes as Raman reporters. It can also be seen that dye 16 produced a more intense SERS spectrum than dye 17, confirming that the selenolates have greater affinity to the gold surface of the HGNs than the thiolates. In general, it was observed that the selenophenes produced stronger SERS spectra than the sulfur substituents when comparing all the reporter molecules and this is consistent with our previous findings [28,29].

Pentamethine dyes, 16 and 17, produced the strongest SERS response, but this was to be expected as we have previously demonstrated that increasing the number of *sp*^2^ carbons in the chalcogenopyrylium backbone causes a significant red-shift in the absorption maximum and a resultant increase in the SERS signal. It is thought that by increasing the number of *sp*^2^ carbons, that a greater displacement along the π-backbone will occur during the vibration, resulting in a greater polarizability and electromagnetic enhancement being experienced [28]. Hence for the pentamethine dyes, not only do they have unique S and Se attachment groups, they also have five *sp*^2^ carbons in their backbone; hence, they demonstrate the most red-shifted absorption maxima (table 1). In fact, their wavelength maxima at 959 nm (dye 16) and 986 nm (dye 17) is very close to coinciding with the laser excitation and as such, a contribution from both molecular resonance and surface enhancement is likely to have led to their exceptional SERS response. Furthermore, as shown in figure 1 and electronic supplementary material, figure S3, all 17 chalcogen dyes produced outstanding SERS signals with HGNs using the 1064 nm hand-held spectrometer. HGNs have previously been prepared with LSPRs up to 1320 nm and when synthesized with a shell thickness greater than 9 nm, they can be used as efficient SERS substrates [31,38,39]. Signal enhancements are observed when the surface plasmon resonance of the substrate matches that of the laser excitation, therefore the fact that the LSPR of the HGNs can be tuned from 500 to 1320 nm, we believe that these chalcogen nanotags could be designed to work with any laser excitation from the visible to the SWIR region. In addition, as previously mentioned, the Raman signals obtained would be significantly enhanced through the SERRS effect, increasing the value and opportunities for the use of these nanotags. See table 1 for the absorption maximum (*λ*_max_) values for the 17 chalcogenopyrylium dyes. Extinction spectra highlighting the wavelength maximum for a selection of the chalcogenopyrylium dyes are provided in electronic supplementary material, figure S4.

**Figure 2.**
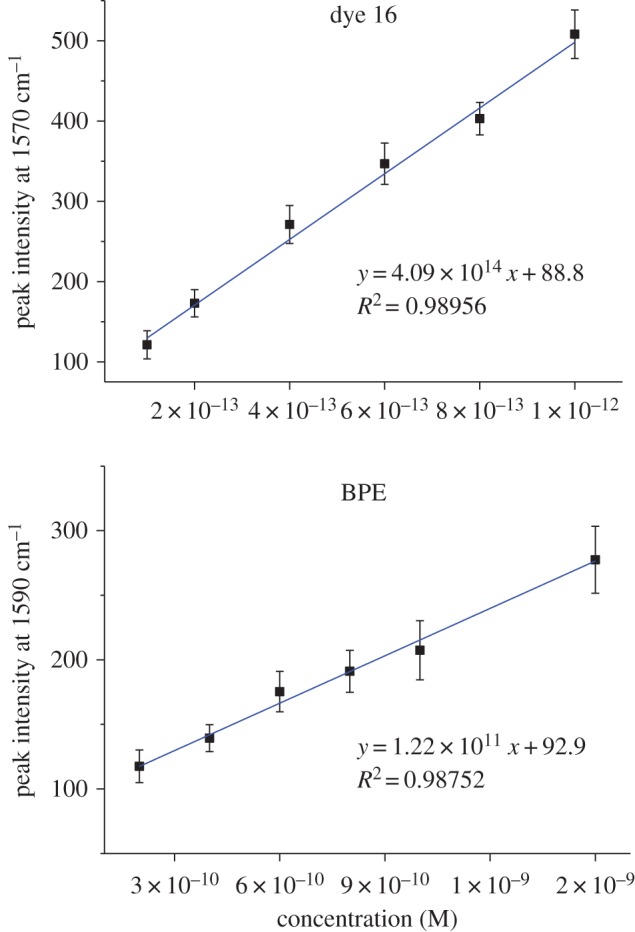
SERS particle dilution studies for chalcogenopyrylium dye 16 plus commercial Raman reporter BPE with HGNs and KCl over the concentration range 2 nM–0.1 pM. A laser excitation of 1064 nm and an exposure time of 7 s were employed in this analysis. Error bars represent 1 s.d. resulting from three replicate samples and five scans of each.

Owing to the remarkable SERS response obtained with the hand-held spectrometer, it was important to investigate the level of sensitivity which could be achieved; therefore, particle dilution studies were conducted in order to determine limits of detection (LODs). Therefore, all 17 chalcogen dyes plus BPE and AZPY with HGNs were analysed over the concentration range 2 nM–0.1 pM. It should be noted that the nanoshells were initially mixed with a Raman reporter at a dye concentration of 10 µM before being diluted with deionized water to the required concentration. The initial particle concentration was 2 nM and subsequent dilutions were made until no signals from the nanotags were observed with the hand-held spectrometer. All experimental conditions were kept the same as those stated previously except an exposure time of 7 s was employed in this analysis. The peak at approximately 1600 cm^−1^ was used to calculate the LOD because it was the most intense peak in the spectrum. Figure 2 and electronic supplementary material, figure S5 show that a linear response was obtained for all the nanotags. The LOD was calculated to be three times the standard deviation (s.d.) of the blank, divided by the gradient of the straight line which can be observed in each of the plots in figure 2 and electronic supplementary material, figure S5. Furthermore, table 1 lists the LOD values with associated s.d. values for each of the chalcogen nanotags plus the LODs obtained for BPE and AZPY analysed using 1064 nm laser excitations.

The pentamethine dyes 16 and 17 produced the best results with extremely low values of 4.6 ± 0.34 fM and 4.8 ± 0.37 fM, respectively. Trimethine dyes 9–15 had LODs from 0.1 to 1.0 pM, while the monomethine dyes 1–8 had LODs from 0.5 to 1.1 pM. However, the commercially available reporters gave the highest LOD values at 0.15 and 0.19 nM for AZPY and BPE, respectively. These are two to three orders of magnitude higher than the monomethine and trimethine chalcogenopyrylium dyes and four to five orders of magnitude higher than the pentamethine chalcogenopyrylium dyes. This demonstrates the superiority of these chalcogenopyrylium nanotags for use with this SWIR laser excitation. Furthermore, all 17 of the chalcogen nanotags were shown to have an LOD value at least one order of magnitude below the commercial ones, with values ranging from 1.1 pM to 4.6 fM. The sensitivity which has been achieved at this laser excitation wavelength is exceptional; however, the fact the results were also obtained using a hand-held spectrometer is unprecedented. These unique nanotags are capable of providing strong SERS signals in the biological window of the SWIR region, but the fact that they provide ultra-low sensitivity with a hand-held device means that these results could provide the basis for future advancements in biomedical and optical applications.

## 2. Conclusion

We have demonstrated that femtomolar sensitivity can be achieved using a hand-held Raman spectrometer incorporating a 1064 nm laser excitation. The combination of chalcogenopyrylium dyes plus HGNs makes ideal SERS nanotags for operating in the SWIR region. These unique tags have demonstrated detection limits two to five orders of magnitude lower than the commercially available ones. In addition, the fact their LSPRs can be tuned well into the infrared, making them flexible across a range of laser wavelengths. Hence, the combination of these chalcogen nanotags plus a hand-held instrument makes for great advancements in terms of the portability and sensitivity required for future applications in homeland security, food safety and biomedical analysis.

## 3. Material and methods

With the exception of dye 15, dyes 1–17 were synthesized as reported in previous publications by Bedics, Kearns and co-workers [28,29]. Additionally, the synthetic procedure for HGNs was also detailed in these publications.

**Scheme 1.**
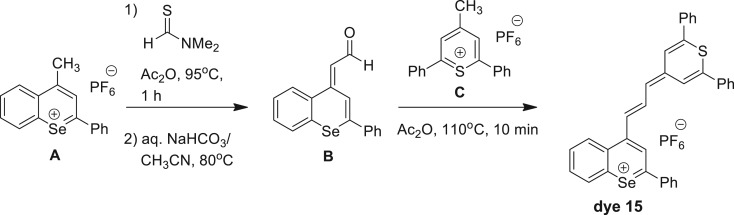
Synthesis of dye 15.

Dye 15 [4-(3-(2,6-diphenyl-4H-thiopyran-4-ylidene)prop-1-enyl)-2-phenylselenobenzo-pyrylium hexafluorophosphate] is new to this subset of chalcogenopyrylium dyes and, as such, the synthesis scheme is provided in scheme 1, with the experimental detail for the synthesis and characterization of this dye along with dyes 16 and 17 being provided in the electronic supplementary material.

### 3.1. Surface-enhanced Raman scattering characterization

Investigation into the properties of the SERS nanotags was carried out by mixing ‘as-prepared’ HGN solution (270 µl; 2.8 nM) with Raman reporter solution (40 µl; 10 µM) and potassium chloride (300 µl; 30 mM). At this excitation wavelength, 19 Raman reporters were analysed, two of which where the commercial reporters BPE and AZPY for comparison. The structures of all Raman reporters analysed can be seen in electronic supplementary material, figure S2. The SERS measurements were performed using a hand-held Snowy Range ‘CBEx’ Raman spectrometer (Laramie, USA) with a diode laser operating at 1064 nm excitation wavelength. The portable spectrometer has the following dimensions 4.5 × 3.125 × 2.25 inches (114 × 79 × 57 mm) and weighs just 773 g (27 oz). It should be noted that experimental set-up S1*a* was employed in this analysis (electronic supplementary material, schematic S1). All the measurements had a 0.05–1 s acquisition time and a laser power operating at 30 mW. Each sample was prepared in triplicate and five scans of each replicate were recorded. All the Raman spectra were background corrected using a multi-point linear fit with zero-levelling mode in Grams software (AI 7.0).

For the SERS particle dilution studies, the optimum conditions were initially used (as stated above) and deionized water was added to obtain subsequent concentrations, over the concentration range of 2 nM–0.1 pM. All other experimental conditions were kept the same as those stated above except an exposure time of 7 s was employed in this analysis. The LOD was calculated to be three times the standard deviation of the blank, divided by the gradient of the straight line. Error bars represent 1 s.d. resulting from three replicate samples and five scans of each, see table 1 for the LOD values for chalcogen dyes 1–17 plus BPE and AZPY adsorbed onto HGNs and electronic supplementary material, figure S5 for associated LOD plots with error bars for dyes 1–15, 17 and AZPY.

A schematic detailing the experimental set-up (electronic supplementary material, schematic S1) plus additional information regarding the preparation of the nanotags are provided in the electronic supplementary material.

## Supplementary Material

Supporting Information

## Data accessibility

Electronic supplementary material available: general experimental details for the synthesis and characterization of dyes 1–17 and HGNs; schematic detailing experimental set-up; figures of extinction spectra for HGNs and selected chalcogenopyrylium dyes; chemical structures for dyes 1–17 and commercial reporters; SERS spectra and LOD plots for dye/HGN nanotags plus commercial reporter/HGN nanotags with 1064 nm excitation. The research data associated with this paper are available at the following link: http://dx.doi.org/10.15129/11368265-f134-4d8b-91d1-0e9b916e7c12.
