# Supplementary material for: Sensitive SERS nanotags for use with a hand-held 1064 nm Raman spectrometer
Source: R Soc Open Sci. 2017 Jul 19;4(7):170422. doi: 10.1098/rsos.170422 (PMC5541563; doi:10.1098/rsos.170422)
Supplement: Supporting Information [file rsos170422supp1.docx]

**Supporting Information**

**Sensitive SERS nanotags for use with a hand-held 1064 nm Raman Spectrometer**

Hayleigh Kearns,^a^ Fatima Ali, ^a^ Matthew A. Bedics, ^b^ Neil C. Shand, ^c^ Karen Faulds, ^a^ Michael R. Detty, ^b^ and Duncan Graham ^a^*

^a^ Department of Pure and Applied Chemistry, Technology and Innovation Centre, University of Strathclyde, 99 George Street, Glasgow, G1 1RD, United Kingdom; Email: duncan.graham@strath.ac.uk

^b^ Department of Chemistry, University at Buffalo, The State University of New York, New York 14260, United States

^c^ Dstl, Porton Down, Salisbury, Wiltshire, SP4 0JQ, United Kingdom

**Experimental**

***Synthesis of dyes***

Dyes 1-17 were synthesised as reported in previous publications by Bedics and Kearns *et al.*(1, 2)

***Dye 15*** [4-(3-(2,6-diphenyl-4H-thiopyran-4-ylidene)prop-1-enyl)-2-phenylselenobenzo-pyrylium hexafluorophosphate] is new to this subset of chalcogenopyrylium dyes and was synthesised as follows:

**Scheme 1.** Synthesis of Dye 15. Figure is reproduced from materials and methods section of manuscript.

4-Methyl-2-phenylselenobenzopyrylium hexafluorophosphate (3, 4) **A** (0.100 g, 0.233 mmol), *N,N*-dimethylthioformamide (59.4 μL, 0.698 mmol) and Ac_2_O (2.0 mL) were combined in a small round bottom flask and heated at 95 °C for 1 h (Scheme 1). After cooling to ambient temperature the solution was diluted with ether. The formed iminium salt was allowed to precipitate in the freezer overnight, and then isolated by filtration to yield a bright orange solid. This solid was dissolved in CH_3_CN (3.0 mL) and satd. aqueous NaHCO_3_ (3.0 mL) was added. This mixture was heated to 80 °C over a 15-min period, and kept at 80 °C for 30 min. After diluting with H_2_O (30 mL) the product was extracted with CH_2_Cl_2_ (3 × 20 mL), dried with Na_2_SO_4_ and purified on SiO_2_ with a CH_2_Cl_2_ eluent (R*_f_* 0.56) to give a yellow oil that was recrystallized in CH_2_Cl_2_/hexanes to yield 66.0 mg (91%) of **B** [(*E*)-2-(2-phenyl-4*H*-selenochromen-4-ylidene)acetaldehyde] as a yellow crystalline solid, mp 89-92 °C: ^1^H NMR [300 MHz, CDCl_3_] δ 10.35 (d, 1 H, *J* = 7.0 Hz), 8.34 (s, 1 H), 8.00-7.97 (m, 1 H), 7.65- 7.57 (m, 3 H), 7.49-7.40 (m, 5 H) 6.53 (d, 1 H, *J* = 6.5 Hz); ^13^C NMR [75.5 MHz, CDCl_3_] δ 189.8, 148.4, 143.2, 138.9, 132.3, 129.9, 129.7, 129.12, 129.08 128.1, 127.1, 126.9, 119.4, 119.1; HRMS (EI) *m/z* 312.0037 (calcd for C_17_H_12_O^80^Se : 312.0048).

4-Methyl-2,6-di(phenyl)thiopyrylium hexafluorophosphate **C** (51.5 mg, 0.126 mmol), aldehyde **B** (43.2 mg, 0.139 mmol), and Ac_2_O (2.0 mL) were combined and heated at 105 °C for 10 min (Scheme 1). The solution was cooled to ambient temperature, diluted with CH_3_CN, and the product precipitated with ether to yield 65.3 mg (74%) of **Dye 15** as a copper bronze solid, mp > 260 °C: λ_max_ (CH_3_CN) = 748 nm (ε 6.1 × 10^4^ M^-1^cm^-1^). Anal. Calcd for C_35_H_25_SSe⋅PF_6_: C, 59.92; H, 3.59. Found: C, 59.74; H, 3.48; HRMS (ESI) *m/z* 557.0853 (calcd for C_35_H_25_S^80^Se^+^ : 557.0837).

***Synthesis of Dye 1*6** - Information is reproduced from publication by Kearns *et al.* (2)

[4-((1E,3E)-5-(2,6-di(selenophen-2-yl)-4H-thiopyran-4-ylidene)penta-1,3-dien-1-yl)-2,6-di(selenophen-2-yl)thiopyrylium hexafluorophosphate]

4-methyl-2,6-di(selenophen-2-yl)thiopyrylium hexafluorophosphate (0.100 g, 0.194 mmol), N-3-(phenylamino)allylidene)benzenaminium hexafluorophosphate (35.7 mg, 96.9 μmol), NaOAc (15.9 mg, 0.194 mmol), AcOH (1.0 mL) and Ac2O (1.0 mL) were combined and heated to 95 °C for 10 min prior to cooling to ambient temperature and diluting with CH3CN (3.0 mL). Ether (5 mL) was added to precipitate the product. Product yield was 61.0 mg (68%) of Dye 16 as a copper-bronze solid, mp 210-212 °C: 1H NMR [500 MHz, CD2Cl2] δ 8.34 (d, 4 H, J = 5.5 Hz), 7.95 (t, 1 H, J = 13.0 Hz), 7.82 (br s, 4 H), 7.60-7.46 (br s, 4 H), 7.44 (t, 4 H, J = 4.5 Hz), 6.75 (t, 1 H, J = 11.0 Hz), 6.52 (d, 2 H, J = 13.0 Hz); λmax (CH2Cl2) 959 nm (ε = 2.7 × 105 M-1cm-1); HRMS (ESI) m/z 776.7717 (calcd for C31H21S280Se4+: 776.7740). Anal. Calcd for C31H21S2Se4⋅PF6: C, 40.54; H, 2.30. Found: C, 40.84; H, 2.51.

***Synthesis of Dye 17*** - Information is reproduced from publication by Kearns *et al.* (2)

[4-((1E,3E)-5-(2,6-di(thiophen-2-yl)-4H-selenopyran-4-ylidene)penta-1,3-dien-1-yl)-2,6-di(thiophen-2-yl)selenopyrylium hexafluorophosphate]

4-Methyl-2,6-di(thiophen-2-yl)selenopyrylium hexafluorophosphate (80.0 mg, 0.171 mmol), N-3-(phenylamino)allylidene)benzenaminium hexafluorophosphate (31.5 mg, 85.6 μmol), NaOAc (14.0 mg, 0.171 mmol), AcOH (2.0 mL) and Ac2O (2.0 mL) were combined and heated to 90 °C for 3 min prior to cooling to ambient temperature and diluting with CH3CN (3.0 mL). Ether (5 mL) was added to precipitate the product. Product yield was 33.1 mg (47%) of Dye 17 as a copper-bronze solid, mp 206-208 °C: 1H NMR [500 MHz, CD2Cl2] δ 8.00 (t, 2 H, J = 11.0 Hz), 7.64-7.63 (m, 8 H), 7.56 (br s, 4 H), 7.20 (t, 4 H, J = 4.5 Hz), 6.82 (t, 1 H, J = 12.0 Hz), 6.65 (d, 2 H, J = 12.5 Hz); λmax (CH2Cl2) 880 nm (ε = 6.6 × 104 M-1 cm-1), 986 nm (ε = 2.6 × 105 M-1 cm-1); HRMS (ESI) m/z 680.8821 (calcd for C31H21S480Se2+: 680.8851). Anal. Calcd for C31H21S4Se2⋅PF6: C, 45.15; H, 2.57. Found: C, 45.24; H, 2.65.

4-Methyl-2,6-di(selenophen-2-yl)thiopyrylium hexafluorophosphate and 4-methyl-2,6-di(thiophen-2-yl)selenopyrylium hexafluorophosphate were synthesised and characterised as reported in a previous publication by Bedics *et at.* (1)

The commercial reporters BPE (1,2-bis(4-pyridyl)ethylene) and AZPY (4,4-azopyridine) were purchased from Sigma-Aldrich.

***Synthesis of HGNs***

The HGNs were synthesised and characterised as reported in a previous publication by Bedics *et al.*(1) The HGNs synthesised in this manner had a concentration of 2.8 nM and a localised surface plasmon resonance at 710 nm (figure S1). The extinction spectrum for the HGNs was acquired using a Perkin Elmer Lambda 35 UV-Vis spectrometer with a wavelength range from 200-1100 nm.

***Preparation of Raman Reporter Solutions***

The chalcogen reporters were prepared as described in references.(1, 2) Dyes 1-17 were dissolved in 2.5 mL of dimethylformamide (DMF) to yield concentrations between 3-5 mM. Subsequent dilutions to 10 µM were prepared in 1:1 ratios of DMF to deionised water. The commercial reporters BPE and AZPY were dissolved in methanol to yield concentrations of 10 mM and subsequent dilutions were prepared in deionised water.

***SERS Characterisation***

The SERS measurements were performed using a hand-held Snowy Range ‘CBEx’ Raman spectrometer (Laramie, USA) with a diode laser operating at 1064 nm excitation wavelength. The portable spectrometer has the following dimensions 4.5 x 3.125 x 2.25 inches (114 x 79 x 57 mm) and weighs just 773 g (27 oz). It should be noted that experimental setup a) was employed in this analysis.


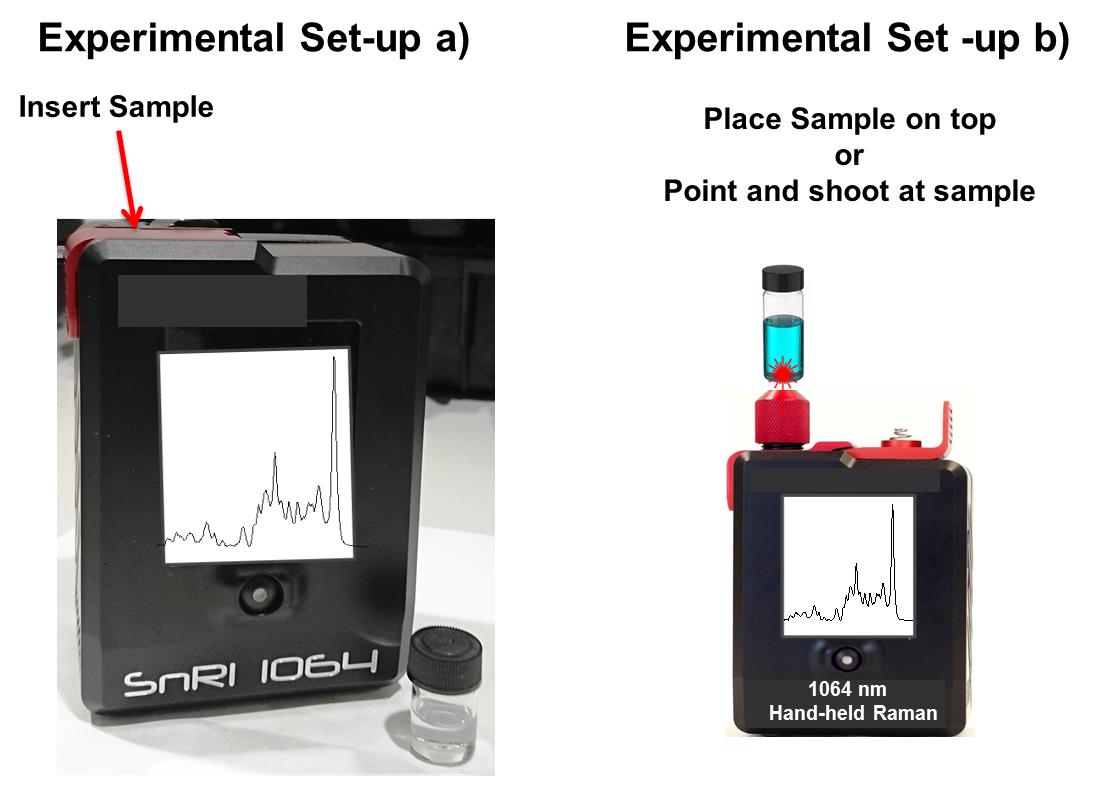


**Schematic S1.** Experimental setups detailing how a sample could be interrogated using a hand-held Snowy Range ‘CBEx’ Raman spectrometer. For set-up a) the sample is placed inside the instrument and analysed whereas for set-up b) the point and shoot tip is used.

**Figure S1.** Extinction spectrum showing the SPR of the HGNs at 710 nm.

**Figure S2.** Chemical structures of the chalcogenopyrylium dyes and commercial reporters used in the SERS experiments.

Figure S3. SERS spectra for chalcogen dyes 1-15, 17 and commercial reporter AZPY (10 µM) analysed with HGNs (SPR recorded at 710 nm) and KCl (30 mM). A laser excitation of 1064 nm and an exposure time of 0.1s were employed in this analysis; unless stated otherwise on the spectra. All spectra have been background corrected.

Figure S4. Extinction spectra for a selection of the chalcogenopyrylium dyes.

**Figure S5.** SERS particle dilution studies for chalcogen dyes 1-15, 17 and commercial reporter AZPY with HGNs and KCl over the concentration range 2 nM to 0.1 pM. A laser excitation of 1064 nm and an exposure time of 7 seconds were employed in this analysis. Error bars represent one standard deviation resulting from 3 replicate samples and 5 scans of each.

**References**

1. Bedics M, Kearns H, Cox JM, Mabbott S, Ali F, Shand NC, Faulds K, Bedics JB, Graham D, Detty MR. Extreme Red Shifted SERS Nanotags. Chemical Science. 2015;**6**:2302-6 (doi:10.1039/C4SC03917C).

2. Kearns H, Bedics MA, Shand NC, Faulds K, Detty MR, Graham D. Sensitive SERS nanotags for use with 1550 nm (retina-safe) laser excitation. Analyst. 2016;**141**(17):5062-5 (doi:10.1039/C5AN02662H).

3. Bodwell JR, Patwardhan BH, Dittmer DC. S-Substituted thiacyclobutenium salts. Journal of Organic Chemistry. 1984;**49**(22):4192-6 (doi:10.1021/jo00196a018).

4. Wizinger R, Angliker H. 244. Contribution to thiopyrylium salts and thiopyrylocyanines. Helvetica Chimica Acta. 1966;**49**(7):2046-55 (doi:10.1002/hlca.660490711).
